# Formose reaction accelerated in aerosol-OT reverse micelles

**DOI:** 10.3762/bjoc.12.262

**Published:** 2016-12-07

**Authors:** Makoto Masaoka, Tomohiro Michitaka, Akihito Hashidzume

**Affiliations:** 1 Department of Macromolecular Science, Graduate School of Science, Osaka University, 1-1 Machikaneyama-cho, Toyonaka, Osaka 560-0043, Japan

**Keywords:** aerosol-OT, formose reaction, hexadecyltrimethylammonium bromide, interfacial layer, reverse micelles, triton X-100, water pool

## Abstract

The formose reaction in reverse micelles of aerosol-OT (AOT), triton X-100 (TX), and hexadecyltrimethylammonium bromide (CTAB) was investigated. Time–conversion data have indicated that the interfacial water layer of AOT reverse micelles is a medium that accelerates formation of glycolaldehyde in the formose reaction. The ^13^C NMR spectra for the products of the formose reaction using formaldehyde-^13^C as starting material are indicative of the formation of ethylene glycol as a major product.

## Findings

The ‘formose reaction’ yields a mixture of sugars and sugar alcohols, called ‘formose’, from formaldehyde by heating under basic conditions. It has been considered that the formose reaction is a possible pathway for sugar formation under prebiotic conditions [1–4]. The formose reaction was first reported by Butlerow in 1861 [5]. Studies on the formose reaction by a number of researchers have revealed that the formose reaction consists of three periods, i.e., the induction period, the sugar formation period, and the sugar degradation period [6]. In the induction period two formaldehyde molecules form glycolaldehyde, which is the rate-determining step of the formose reaction. It is known that glycolaldehyde acts as a cocatalyst. Thus, when the concentration of glycolaldehyde reaches a certain level, formaldehyde is consumed rapidly to form a complicated mixture of sugars predominantly via aldol reaction and aldose–ketose transformation in the sugar formation period. When formaldehyde is consumed quantitatively, the reaction mixture turns yellow. In the sugar degradation period, the sugars formed are decomposed dominantly through cross-Cannizzaro reaction and retro-aldol reaction to form a more complicated reaction mixture. The control of the formose reaction was investigated by several research groups [7–16], but it has been still a great challenge to form useful sugars by the formose reaction. Recently, since we focus on the use of reaction media of nanometer scale for the control of the formose reaction [17], we have carried out the reaction in reverse micelles and found that the formose reaction in reverse micelles does not show the induction period. This letter thus describes the formose reaction accelerated in reverse micelles.

**Scheme 1:**
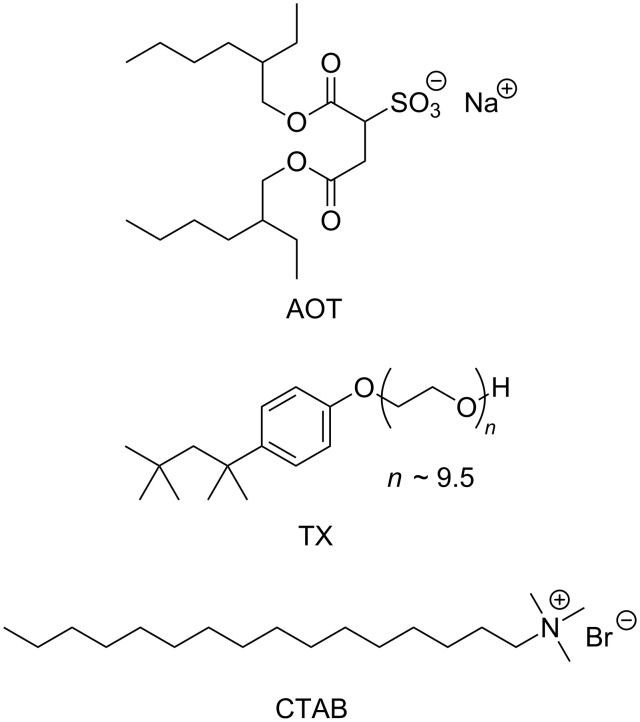
Structures of AOT, TX, and CTAB.

In this study, we have used reverse micelles of anionic aerosol OT (AOT), nonionic triton X-100 (TX), and cationic hexadecyltrimethylammonium bromide (CTAB) (Scheme 1). Before investigating the formose reaction, the sizes of water pools of the reverse micelles formed from the surfactants were evaluated under several conditions (see Supporting Information File 1). In the cases of AOT and CTAB, the radius of the water pools (*R*_w_) was evaluated by dynamic light scattering, whereas, in the case of TX, *R*_w_ was estimated by the fluorescence quenching technique. As can be seen in Supporting Information File 1, Figure S1, *R*_w_ for AOT and CTAB is almost proportional to the molar ratio of water to surfactant, *w* (= [water]/[surfactant]), independent of the temperature. At *w* = 10, AOT and CTAB reverse micelles contain water pools of *R*_w_ ≈ 3 and 2 nm, respectively. On the other hand, *R*_w_ for TX is nearly constant at ca. 1 nm independent of *w* and temperature.

**Figure 1:**
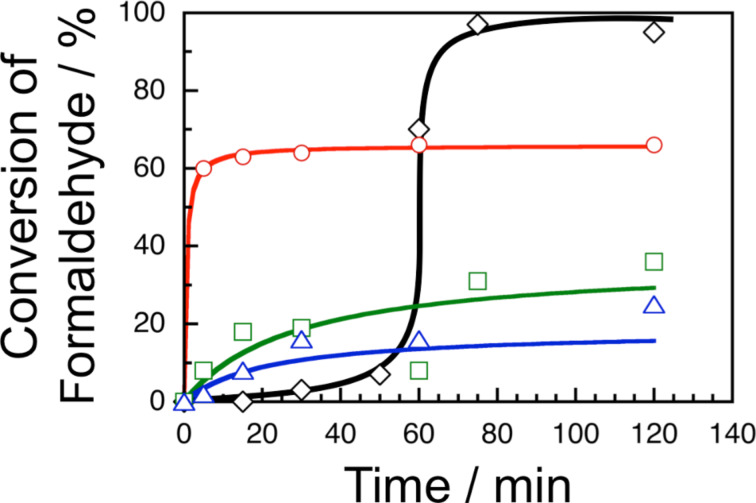
Time–conversion plots for formose reactions in an aqueous solution (black) and in water pools of AOT (red), TX (green), and CTAB reverse micelles (blue) at 60 °C. The curves are drawn as a guide for the eye.

The formose reactions were carried out at 60 °C by mixing a 100 mM solution of a surfactant in isooctane or in a mixed solvent of isooctane and 1-hexanol (5:1, v/v) and an aqueous solution containing 200 mM formaldehyde and 20 mM calcium hydroxide to adjust *w* to 10. After predetermined times, aliquots of the reaction mixture were taken and neutralized with 100 mM hydrochloric acid to terminate the reaction. The remaining unreacted formaldehyde was then extracted with water several times. Using the combined water phase, the concentration of unreacted formaldehyde was determined by the acetylacetone method [18–19] to evaluate the conversion of the formose reaction. Figure 1 demonstrates the time–conversion plots for the formose reactions in water pools of AOT, TX, and CTAB reverse micelles and that in an aqueous solution. In the formose reaction in an aqueous solution, i.e., a reference experiment, after an induction period of 0–50 min, the conversion commenced to increase rapidly and reached a quantitative conversion at 75 min. In the formose reactions in AOT, TX, and CTAB reverse micelles, on the other hand, no induction period was observed, indicative of acceleration of the formation of glycolaldehyde. The acceleration was most remarkable in the case of AOT reverse micelles. It is noteworthy that the conversion levelled off and did not reach a quantitative yield. The saturated conversions were ca. 65, ca. 35, and ca. 25% for AOT, TX, and CTAB reverse micelles, respectively [20]. On the basis of these observations, AOT provides the most efficient medium for the formose reaction among the surfactants examined in this study.

**Figure 2:**
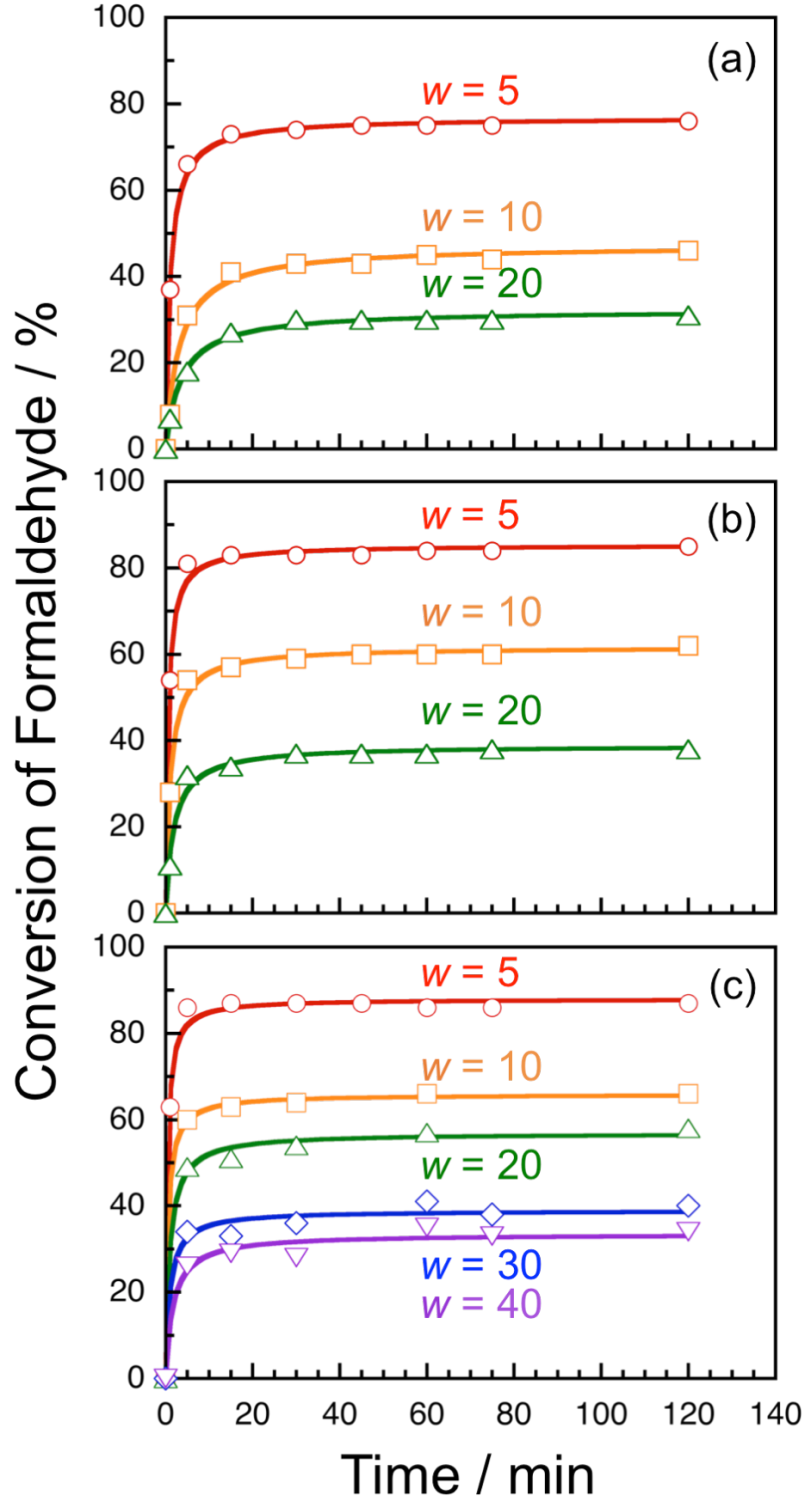
Time–conversion plots for formose reactions in AOT reverse micelles of different *w* at 30 (a), 45 (b), and 60 °C (c). Curves indicate the best fits using Equation 1.

The formose reaction in water pools of AOT reverse micelles were investigated at varying *w* and temperatures. Figure 2 shows time–conversion plots for AOT reverse micelles of different *w* at 30, 45, and 60 °C. This figure indicates that there is no induction period at all the *w* and temperatures examined. It should be noted here that the saturated conversion is lower at a larger *w*. Given a second order reaction for formaldehyde, the time–conversion plots were analyzed by

**Figure 3:**
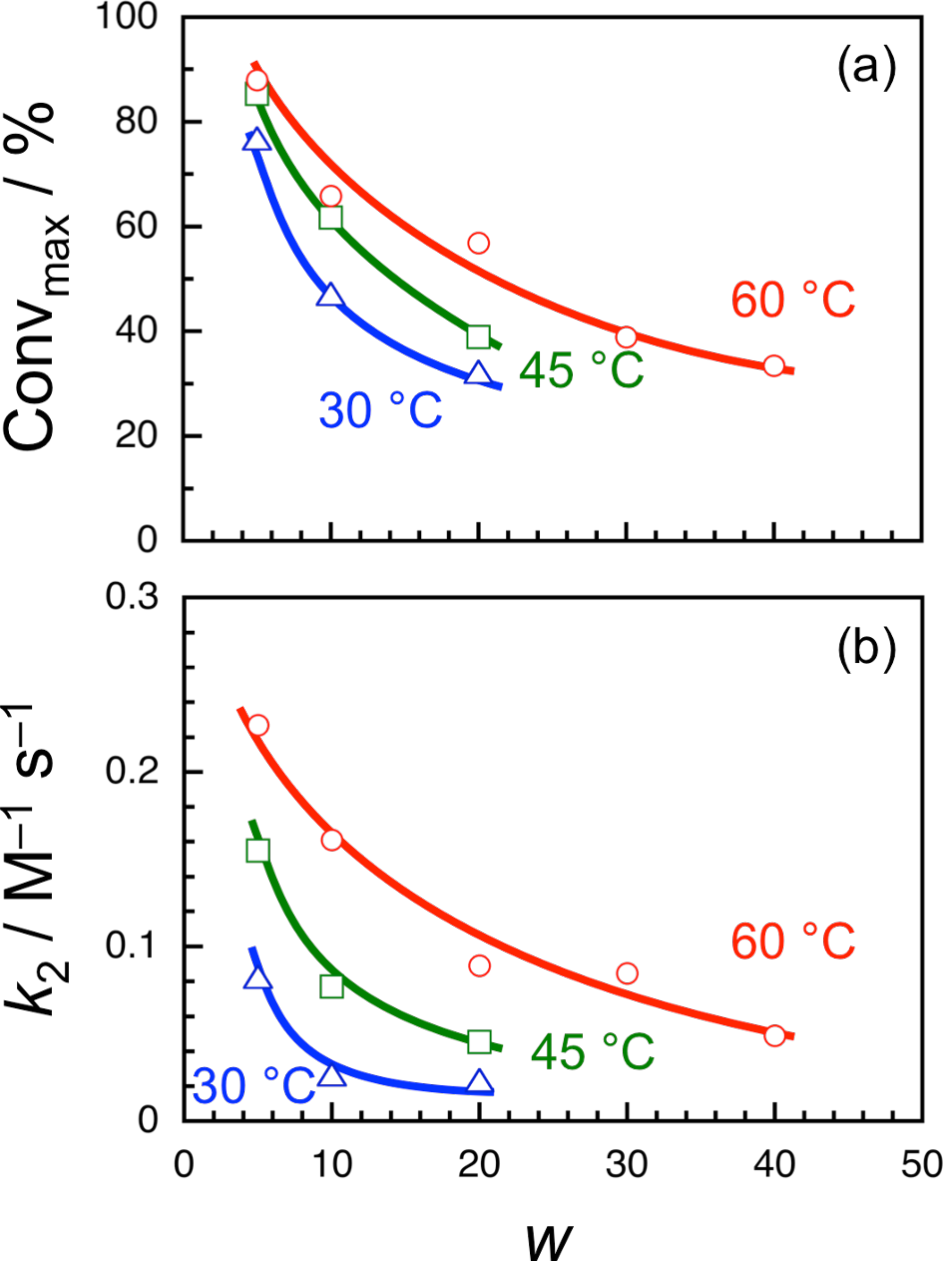
Conv_max_ and *k*_2_ as function of *w* for formose reactions in water pools of AOT reverse micelles at 30 (triangle), 45 (square), and 60 °C (circle). The curves are drawn as a guide for the eye.

[1]
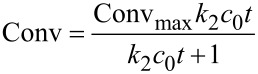


where Conv and Conv_max_ denote the conversion at time *t* and the saturated conversion, respectively, and *k*_2_ and *c*_0_ are the *pseudo*-second-order rate constants and the initial concentration of formaldehyde (= 200 mM), respectively [21]. As can be seen in Figure 2, the best fitted curves agree well with the experimental data. Values of Conv_max_ and *k*_2_ obtained from the fitting were plotted in Figure 3 against *w* at different temperatures. This figure indicates that both Conv_max_ and *k*_2_ decrease with increasing *w* at all the temperatures examined, indicating that the formose reaction proceeds more efficiently in AOT reverse micelles of a smaller *w*. Since the formose reaction did not proceed in aqueous solutions at 30 °C and the same concentrations of formaldehyde and calcium hydroxide, it is concluded that the water pools of AOT reverse micelles act as an effective medium for the formose reaction.

Here it is worth to discuss about the *R*_w_ of AOT reverse micelles of *w* = 5. As can be seen in Supporting Information File 1, Figure S1, *R*_w_ is ca. 1 nm for water pools of AOT reverse micelles of *w* = 5. Even though a water pool of *R*_w_ ≈ 1 nm contains one formaldehyde molecule on average under the conditions used (i.e., at 200 mM formaldehyde), the values of Conv_max_ and *k*_2_ were the largest at *w* = 5. These observations indicate that the contents are frequently exchanged between water pools of AOT reverse micelles during the reaction.

On the interface between the surfactant molecules and the water phase in the reverse micelles, there is a layer of water molecules with restricted mobility, because of hydration of the hydrophilic group of the surfactant (i.e., the surfactant head) [22–23]. It is likely that the thickness of the layer of restricted water is approximately several nm [24–25]. The water molecules in the interfacial layer are more polar than those in the bulk water phase because of polarization caused by hydration of the surfactant head. Since *R*_w_ of AOT reverse micelles is practically proportional to *w* (Figure S1 in Supporting Information File 1), the fraction of water molecules in the interfacial layer increases with decreasing *w*. As can be seen in Figure 3, Conv_max_ and *k*_2_ values are larger at a smaller *w*. It can be thus concluded that the interfacial layer of restricted water provides an efficient medium for the formose reaction.

**Figure 4:**
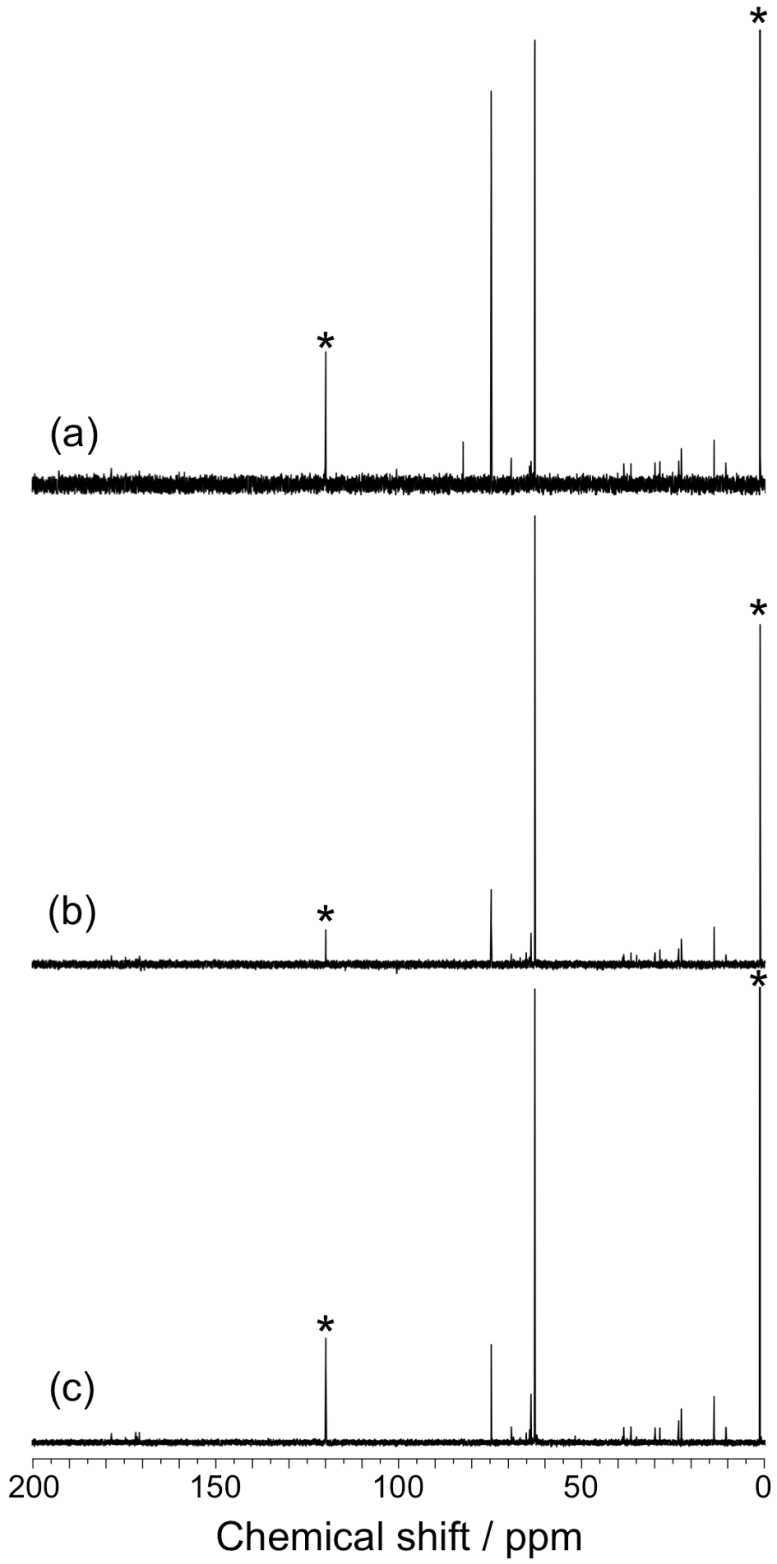
^13^C NMR spectra for the products of the formose reaction carried out in AOT reverse micelles of *w* = 5 using formaldehyde-^13^C at 35 (a), 45 (b), and 60 °C for 60 min (c). Asterisks represent the signals of acetonitrile, i.e., the internal standard.

It is important to characterize the product of the formose reaction in AOT reverse micelles. However, it was not possible to purify the product because the reaction mixture contained a large amount of AOT. After a large fraction of AOT was removed, the mixture obtained was measured by high-performance liquid chromatography, NMR spectroscopy, and mass spectrometry, but no signals ascribable to the products, i.e., sugars or sugar alcohols, were observed because of the residual AOT. The formose reaction was thus carried out in AOT reverse micelles of *w* = 5 using formaldehyde-^13^C as starting material at 30, 45, and 60 °C for 60 min, and the product was characterized by ^13^C NMR after removal of a large fraction of AOT (see Supporting Information File 1). Figure 4 compares ^13^C NMR spectra for the products of the formose reaction carried out using formaldehyde-^13^C at 30, 45, and 60 °C. In these spectra, signals at 1.5 and 119.5 ppm are due to the methyl and nitrile carbons in acetonitrile, the internal standard, respectively. All the spectra contain two intense signals at ca. 63 and 75 ppm, although the ratios of signal intensities are different at different temperatures. The signal at ca. 63 ppm can be assigned to ethylene glycol, which may be derived from glycolaldehyde through Cannizzaro reaction. (We could not assign the signal at ca. 75 ppm comparing to signals for various sugar and sugar alcohols.) On the basis of these spectra, we can conclude that ethylene glycol is formed as a major product.

In summary, the formose reaction in reverse micelles of AOT, TX, and CTAB was investigated. The formose reaction in reverse micelles did not show the induction period, which is shown in the conventional formose reaction, indicating that the formation of glycolaldehyde was accelerated in the reverse micelles. AOT was the most effective among the surfactants examined. The values of Conv_max_ and *k*_2_ were larger at smaller *w*, indicating that the interfacial water layer is a medium that accelerates the formation of glycolaldehyde in the formose reaction. The ^13^C NMR spectra for the products of the formose reaction using formaldehyde-^13^C were indicative for the formation of ethylene glycol as a major product.

## Supporting Information

File 1 Experimental section and characterization of reverse micelles.

## References

[1] Gardner P M, Winzer K, Davis B G (2009). Nat Chem.

[2] Harman C E, Kasting J F, Wolf E T (2013). Origins Life Evol Biospheres.

[3] Kebukawa Y, Kilcoyne A L D, Cody G D (2013). Astrophys J.

[4] Kebukawa Y, Cody G D (2015). Icarus.

[5] Butlerow A (1861). Justus Liebigs Ann Chem.

[6] Mizuno T, Weiss A H (1974). Adv Carbohydr Chem Biochem.

[7] Shigemasa Y, Nagae O, Sakazawa C, Nakashima R, Matsuura T (1978). J Am Chem Soc.

[8] Shigemasa Y, Kawahara M, Sakazawa C, Nakashima R, Matsuura T (1980). J Catal.

[9] Shigemasa Y, Akagi S, Nakashima R, Saito S (1980). Carbohydr Res.

[10] Shigemasa Y, Hamada T, Hirabayashi M, Waki E, Nakashima R, Harada K, Takeda N, Suzuki M (1981). Chem Lett.

[11] Shigemasa Y, Oogaki K, Ueda N, Hakashima R, Harada K, Takeda N, Suzuki M, Saito S (1982). J Carbohydr Chem.

[12] Shigemasa Y, Sasaki Y, Ueda N, Nakashima R (1984). Bull Chem Soc Jpn.

[13] Shigemasa Y, Ueda T, Saimoto H (1990). Bull Chem Soc Jpn.

[14] Matsumoto T, Komiyama M, Inoue S (1980). Chem Lett.

[15] Matsumoto T, Inoue S (1982). J Chem Soc, Perkin Trans 1.

[16] Matsumoto T, Yamamoto H, Inoue S (1984). J Am Chem Soc.

[17] Hashidzume A, Fujimoto T, Masaoka M, Sanada Y, Sato T (2010). Kobunshi Ronbunshu.

[18] Available from: http://www.maff.go.jp/nval/kijyun/pdf/ST06130.PDF

[19] Maruo Y Y, Nakamura J, Uchiyama M (2008). Talanta.

[R20] 20At present, we are not sure why the formose reaction in reverse micelles exhibits a saturated conversion. This may be because the catalyst is deactivated in the first 20–30 min.

[R21] 21The formose reaction starts with the formation of glycolaldehyde from two formaldehyde molecules, which is the rate-determining step in conventional formose reaction. It is thus reasonable to analyze the time-conversion data based on a second-order reaction as a first approximation. Since the conversion does not reach 100% for all the cases examined in the formose reaction in the presence of AOT reverse micelles, we modified the conventional equation of second-order reaction to obtain Equation 1.

[22] Hasegawa M, Sugimura T, Suzaki Y, Shindo Y, Kitahara A (1994). J Phys Chem.

[23] Hasegawa M, Sugimura T, Shindo Y, Kitahara A (1996). Colloids Surf, A.

[24] Pieniazek P A, Lin Y-S, Chowdhary J, Ladanyi B M, Skinner J L (2009). J Phys Chem B.

[25] Chowdhary J, Ladanyi B M (2009). J Phys Chem B.

