# Supplementary material for: Formose reaction accelerated in aerosol-OT reverse micelles
Source: Beilstein J Org Chem. 2016 Dec 7;12:2663–7. doi: 10.3762/bjoc.12.262 (PMC5238542; doi:10.3762/bjoc.12.262)
Supplement: File 1 — Experimental section and characterization of reverse micelles. [file Beilstein_J_Org_Chem-12-2663-s001.pdf]

Supporting Information

for

Formose reaction accelerated in aerosol-OT reverse micelles

Makoto Masaoka, Tomohiro Michitaka and Akihito Hashidzume*

Address: Department of Macromolecular Science, Graduate School of Science, Osaka University, 1-1 Machikaneyama-cho, Toyonaka, Osaka 560-0043, Japan

Email: Akihito Hashidzume - hashidzume@chem.sci.osaka-u.ac.jp

*Corresponding author

Experimental section and characterization of reverse micelles

Experimental section

Materials

AOT was purchased from Tokyo Chemical Industry and used without further purification. TX and CTAB were purchased from Wako Pure Chemical Industries and were used without further purification. An aqueous solution of formaldehyde (36 wt %) and calcium hydroxide were purchased from Sigma-Aldrich Japan. Paraformaldehyde-^{13}C (99 atom % ^{13}C) was purchased from Taiyo Nippon Sanso. Isooctane and 1-hexanol were purchased from Wako Pure Chemical Industries and used without further purification. 3,7-Diamino-2,8-dimethyl-5-phenylphenazinium chloride (safranine T), used as a fluorescent probe, was purchased from Tokyo Chemical Industry. Potassium bromide, used as a quencher, was purchased from Sigma-Aldrich Japan. Water was purified by a Millipore Milli-Q system. Other reagents were used without further purification.

Measurements

UV–vis spectra were recorded on a JASCO V-550 spectrometer.

Dynamic light scattering (DLS) measurements were performed using an ALV/SLS/DLS-5000 light scattering instrument equipped with an ALV-5000 multiple τ digital correlator and a Nd:YAG laser operating at 532 nm at 30, 45, and 60 °C. The intensity autocorrelation functions $\{g^{(2)}(t)\}$ obtained were analyzed by the CONTIN algorithm to estimate the spectrum $\{A(\tau)\}$ of the relaxation time (τ) at each scattering angle or the magnitude of the scattering vector (k) expressed as

$$k = \frac{4\pi n_0}{\lambda} \sin\left(\frac{\theta}{2}\right) \tag{S1}$$

where n_0 is the refractive index of the solvent, and λ is the wavelength of the incident light. The n_0 value of isooctane at 20 °C was used for all analyses. The hydrodynamic radius R_H can be calculated by

$$R_H = \frac{k_B T}{6\pi\eta_0} \left(\lim_{k \to 0} \frac{\Gamma}{k^2}\right) \tag{S2}$$

where $k_B T$ is the Boltzmann constant multiplied by the absolute temperature, and η_0 is the solvent viscosity, which was determined for isooctane by viscometry using an Ubbelohde-type viscometer.

Fluorescence spectra for safranine T were recorded with excitation at 520 nm on a Hitachi F-4500 fluorescence spectrometer at 30, 45, and 60 °C. The slit widths for excitation and emission sides were kept at 2.5 nm during measurements.

^{13}C NMR spectra were measured on a JEOL JNM ECA500 using D_2O as a solvent at 25 °C. Chemical shifts were referenced to the signal of the methyl carbon of acetonitrile, i.e., the internal standard (δ = 1.47 ppm).

Formose reaction

A typical procedure of the formose reaction in water pools of reverse micelles is described below. An aqueous solution (1.8 mL) containing 200 mM formaldehyde and 20 mM calcium hydroxide was added to a solution of AOT in isooctane (100 mM, 100 mL) to adjust *w*. The mixture was warmed with a water bath thermostated at 60 °C. After predetermined times, aliquots of the reaction mixture (10 mL) were taken out by a syringe, and the reaction was terminated by rapid cooling with an ice water bath followed by neutralization with aqueous HCl (2.0 M, 0.2 mL). Water (10 mL) was added to the mixture to extract the remaining formaldehyde, and then the mixture was centrifuged at 4000 rpm for 15 min. The aqueous layer obtained was washed with dichloromethane (10 mL) once. Using the clear aqueous layer obtained, the concentration of formaldehyde was determined by the acetylacetone method [s1,s2].

As a reference experiment, the formose reaction was also carried out in aqueous media as follows. An aqueous solution of formaldehyde (200 mM, 18 mL) and calcium hydroxide (0.027 g, 3.6×10^{-4} mol) were placed in a 50 mL recovery flask. The flask was warmed with a water bath thermostated at 60 °C. After predetermined times, aliquots of the reaction mixture (1.0 mL) were taken out by a syringe, and the reaction was terminated by rapid cooling with an ice water bath followed by neutralization with aqueous HCl (2.0 M, 0.2 mL). Water (ca. 40 mL) was added to the mixture, and then the concentration of formaldehyde was determined by the acetylacetone method [s1,s2].

Formose reaction in AOT reverse micelles using formaldehyde-^{13}C

An aqueous solution of $H^{13}CHO$ was prepared by dissolving paraformaldehyde-^{13}C (50.2 mg, 1.62 mmol of $H^{13}CHO$) in water (8 mL) at 40 °C overnight. The

concentration of $H^{13}CHO$ was determined to be 180 mM by the acetylacetone method.

A solution of AOT (200 mM) in isooctane (100 mL) and the aqueous solution of formaldehyde-^{13}C (180 mM, 1.8 mL) were mixed to form an microemulsion. $Ca(OH)_2$ (15 mg, 0.20 mmol) was then added to a flask containing the microemulsion, and the solution was stirred at 30, 45, or 60 °C in a water bath for 60 min. Hydrochloric acid (100 mM, 1 mL) was added to the reaction mixture to quench the reaction. The volatile fractions were evaporated to obtain a pale yellow residue. Water (4 mL) and chloroform (60 mL) was then added to the residue to obtain a cloudy emulsion. The heterogeneous mixture was centrifuged at 4000 rpm for 2 h. The phases were separated and water (2 mL) was added to the organic phase. The cloudy emulsion was centrifuged again at 4000 rpm for 2 h. The water layers obtained were combined, and the aqueous solution was washed with $CHCl_3$ (2 × 50 mL) by centrifugation at 4000 rpm for 10 min twice. The water fraction finally obtained was evaporated. The residue was dried under reduced pressure. The yields were 153, 168, and 173 mg for the reaction at 30, 45, and 60 °C, respectively.

Characterization of Reverse Micelles

Before the study of formose reaction, the sizes of the water pools of the reverse micelles formed from the surfactants were evaluated under several conditions. In the cases of AOT and CTAB, since the hydrophilic groups are small enough compared to the reverse micelles, R_w was calculated from the hydrodynamic radii of water-free reverse micelles and those containing water pools ($R_{H,0}$ and R_H, respectively) as

$$R_w = R_H - R_{H,0} \tag{S3}$$

Here $R_{H,0}$ and R_H were measured by DLS at different temperatures and w (= [water]/[surfactant]) [s3,s4]. Figure S1 shows R_w as a function of w at 30, 45, and 60

°C. In both cases of AOT and CTAB, R_w is almost proportional to *w* independent of temperature. These observations indicate that the area per surfactant molecule (i.e., AOT and CTAB) is almost constant independent of *w* and the temperature in the whole range examined. At $w = 10$, AOT and CTAB reverse micelles contain water pools of $R_w \approx 3$ and 2 nm, respectively. On the other hand, since TX possesses an oligo(ethylene glycol) chain as the hydrophilic group, which is larger compared to the hydrophobic nonylphenyl group, it is not possible to determine R_w from DLS data unlike the cases of AOT and CTAB. Thus, apparent values of R_w were estimated by the steady state fluorescence quenching technique using a pair of water soluble fluorophore and quencher, i.e., safranin T and potassium bromide [s5]. When both the fluorophore and quencher are dissolved in water pools of reverse micelles, the fluorescence from the fluorophore will be quenched. At a constant fluorophore concentration, the fluorescence intensity (*I*) decreases with increasing the quencher concentration ([Q]). Given a Poisson distribution of the quencher molecules between water pools and static quenching, there is a relationship between *I* and [Q]:

$$\ln (I_0/I) = [\mathrm{Q}]/[\mathrm{WP}] \qquad \text{(S4)}$$

where I_0 denotes the fluorescence intensity in the absence of the quencher and [WP] denotes the molar concentration of water pools. Assuming that all the water molecules added form spherical water pools of the density of unity, apparent R_w values are calculated by

$$R_\mathrm{w} = \left(\frac{3V_\mathrm{w}}{4\pi N_\mathrm{A} [\mathrm{WP}] V_\mathrm{soln}} \right)^{-1/3} \qquad \text{(S5)}$$

to be compared with those for AOT and CTAB reverse micelles. Here V_w and V_{soln} denote the volumes of the water added and solution, respectively. As can be seen in Figure S1, R_w for the water pools of TX reverse micelles is nearly constant at ca. 1 nm independent of *w* and temperature.

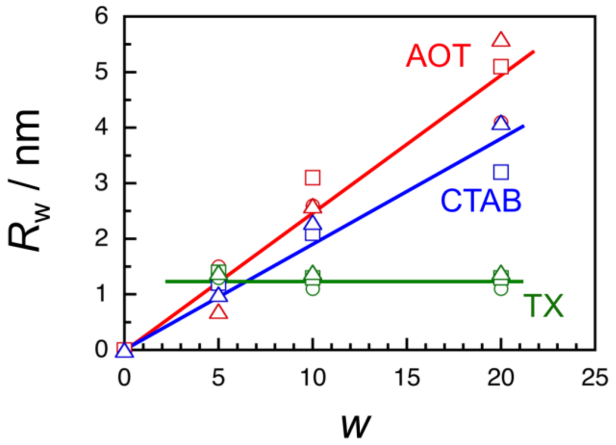

Figure S1: R_w as a function of *w* for AOT (red), TX (green), and CTAB reverse micelles (blue) at 30 (circle), 45 (square), and 60 °C (triangle).

References

s1. http://www.maff.go.jp/nval/kijyun/pdf/ST06130.PDF.

s2. Maruo, Y. Y.; Nakamura, J.; Uchiyama, M. *Talanta* **2008**, *74*, 1141-1147.

s3. Dokter, A. M.; Woutersen, S.; Bakker, H. J. *J. Chem. Phys.* **2007**, *126*, 124507.

s4. Mitra, R. K.; Sinha, S. S.; Pal, S. K. *Langmuir* **2008**, *24*, 49-56.

s5. Nandi, S.; Ghosh, S. K.; Bhattacharya, S. C. *Colloids Surf., A* **2005**, *268*, 118-123.
